# The future digital outpatient clinic for movement disorders: from technological innovation to ethical and systemic implementation—a position paper

**DOI:** 10.1007/s00702-026-03167-3

**Published:** 2026-05-14

**Authors:** Thomas Koeglsperger, Matthias Höllerhage, Jan Kassubek, Dirk Woitalla, Jürgen Winkler, Jochen Klucken

**Affiliations:** 1 Department of Neurology, LMU University Hospital, LMU Munich, Munich, Germany; 2 Department of Translational Brain Research, German Center for Neurodegenerative Diseases (DZNE), Munich, Germany; 3 Department of Neurology, Hannover Medical School, Carl-Neuberg-Str. 1, 30625 Hannover, Germany; 4 Department of Neurology, University Hospital Ulm, Ulm, Germany; 5 Department of Neurology, St. Josef-Krankenhaus Kupferdreh, Essen, Germany; 6 Department of Molecular Neurology, University of Erlangen, Erlangen, Germany; 7 Digital Medicine, Luxembourg Centre for Systems Biomedicine (LCSB), Centre Hospitalier du Luxembourg, Université du Luxembourg, Esch-Sur- Alzette, Strassen, Luxembourg, Luxembourg

**Keywords:** Digital Health, Telemedicine, Wearables, Remote Monitoring, Movement Disorders, Parkinson's Disease

## Abstract

The digital transformation of healthcare is rapidly reshaping neurology, particularly in the field of movement disorders, where continuous monitoring, long disease trajectories, and complex multimodal care create a high demand for innovative solutions. Wearable sensors, digital diagnostics, app-based therapeutics, and integrated hybrid care networks and digital supported care pathways promise earlier diagnosis, personalized treatment and care management, and improved long-term outcomes. However, real-world implementation in managed outpatient care remains fragmented and faces major barriers beyond pure technological feasibility. This position paper critically reviews the current state of digital technologies in movement disorder care, identifies key systemic, ethical, and economic roadblocks, and proposes a pragmatic roadmap toward a realistic and ethically sound digital outpatient clinic. We argue that the future digital clinic will not be defined solely by technical progress, but by how consciously healthcare systems integrate digital tools to hybrid care solutions while preserving human-centered, equitable, and evidence-based care.

**Supplementary Information:**

The online version contains supplementary material available at https://doi.org/10.1007/s00702-026-03167-3.

## Introduction

Movement disorders, particularly Parkinson’s disease (PD) and related syndromes, represent a growing public health burden due to aging populations, prolonged disease courses, and increasing demands for specialized longitudinal care (Su et al. 2025). At the same time, outpatient neurology faces workforce shortages, limited consultation time, and rising complexity of therapeutic options. Digital health technologies and digital medical devices have therefore been widely promoted as a potential solution to many structural deficits in movement disorder care (Boers et al. 2025; Falkenburger et al. 2025; Fomo et al. 2025; Mejia et al. 2025).

Continuous remote monitoring, algorithm-based diagnostics, and digital therapeutics promise to shift care from episodic clinic visits toward longitudinal, data-driven management (Liu et al. 2022; Yang et al. 2022; Raschka et al. 2025; Sander et al. 2025). However, despite rapid technological progress, the translation of these innovations into routine outpatient care has been limited (Sapienza et al. 2024). Many digital solutions remain isolated pilot projects, and their integration into existing clinical workflows, reimbursement systems, and ethical frameworks is inconsistent. This position paper addresses early adopters, clinical leaders, healthcare decision-makers, and policy stakeholders. Its aim is not to celebrate technological innovation per se, but to provide a clinically grounded, system-oriented, and ethically reflective analysis of how a future digital outpatient clinic for movement disorders could—and should—realistically be shaped. We argue that failure to actively shape this transformation risks increasing fragmentation, loss of clinical guideline and leadership to non-medical actors, and widening inequalities in access to care.

## Conceptual framework: confrontation with reality

Digital transformation in movement disorder care operates within a multidimensional tension field that can be conceptualized along three core axes: data protection, financing, and methodological or human readiness to adopt digital techniques. These dimensions interact and often constrain each other.

First, data protection represents a central legal and societal boundary, particularly in Europe. Movement disorder care increasingly relies on continuous personal data streams, including motor activity, sleep, speech, and potentially prodromal markers. The protection of highly sensitive health data is not a technical detail but a prerequisite for trust and legitimacy of digital care.

Second, quality assessment of digital healthtech solutions linked to financing and reimbursement determine whether innovations remain academic projects or become sustainable components of routine care (Boers et al. 2025). Many digital tools address long-term outcomes such as disease progression, autonomy, or hospital avoidance, while most healthcare systems still primarily reimburse short-term clinical encounters and procedures.

Third, methodological readiness includes both the scientific validation of digital tools and the willingness and competence of patients and healthcare professionals to use them, as well as policy maker to implement them (Paccoud et al. 2024b). Digital illiteracy, lack of training, and professional skepticism remain major limiting factors. Digital transformation therefore confronts healthcare systems not only with technical questions but with deeply human, cultural, and organizational challenges (Paccoud et al. 2024a).

## State of the art in digital movement disorder care

### Digital diagnostics

Digital health technologies have transformed diagnostic assessment in movement disorders by enabling continuous, objective, and remote evaluation of cardinal motor features such as tremor, bradykinesia, gait impairment, and motor fluctuations. Wearable inertial sensors—most commonly accelerometers and gyroscopes embedded in smartwatches and body-worn devices—facilitate long-term passive monitoring of movement patterns in daily life, capturing symptom variability that often eludes episodic clinic assessments (Virameteekul et al. 2025). Recent studies demonstrate that these systems can distinguish patients with PD from healthy controls or atypical Parkinsonian syndromes and track changes in gait dynamics, tremor prevalence, and motor decline over time, reflecting both disease severity and progression (Kirk et al. 2024; Sidoroff et al. 2026; Teckenburg et al. 2026). For example, multicenter observational data from research-grade wearables paired with smartphone task performance revealed significant differences in early PD participants compared to controls across multiple motor domains during free-living assessment periods (Adams et al. 2023; Sander et al. 2025). In parallel, smartphone-based applications augment motion data with guided active tests and contextual symptom diaries. Systematic mapping of movement disorder apps has identified over a hundred tools designed for momentary symptom evaluation, passive tracking, or structured motor tasks—most frequently targeting gait, tremor, and fine motor dexterity (Willemse et al. 2024). However, the integration of innovative diagnostic technologies into healthcare procedures proving their clinical utility remains spares (Sapienza et al. 2024). Moreover, hybrid frameworks using commercial consumer devices show good feasibility for remote monitoring protocols, yielding motor indicators that correlate with traditional clinical scales such as the MDS-UPDRS, albeit with variable strength depending on the symptom assessed (Sigcha et al. 2023).

A key conceptual distinction in digital diagnostics lies between patient-centered tools, which aim to empower self-monitoring and engagement with symptom information, and physician-centered systems, which deliver quantitative digital biomarkers and clinician-facing decision support (Virameteekul et al. 2025). The former prioritizes usability and broad accessibility, often through intuitive interfaces and minimal patient burden. The latter focus on analytical rigor, integrating algorithmic feature extraction and longitudinal data visualization to support clinical interpretation and therapeutic decision-making. Shared platforms that synthesize wearable sensor streams with smartphone-based active tests and patient-reported outcomes are increasingly explored as comprehensive digital phenotypes of motor function (Bacanoiu et al. 2025). Despite promising correlations with established clinical measures, real-world implementation faces critical challenges. Usability studies highlight that device discomfort, technical complexity, and interface limitations can undermine long-term adherence among patients with PD, particularly in older or cognitively impaired individuals (Ma et al. 2025).

Additionally, variable adherence to wearing devices year-long—influenced by battery life, data transfer issues, and symptom burden—points to the need for more robust and user-friendly designs (Maas et al. 2024). From a measurement standpoint, some symptom domains (e.g., gait variability or finger-specific impairment) remain difficult to capture accurately using wrist-only sensors, suggesting that multi-site sensing strategies or task-specific protocols may be necessary for richer diagnostic fidelity. In summary, while wearables, smartphone applications, and ambient monitoring systems hold significant promise for improving the granularity and ecological validity of movement disorder diagnostics, their translation into routine clinical practice will require further validation, better integration into clinical workflows, and solutions to challenges related to long-term adherence and interpretability. A key limitation remains the absence of a true ground truth for validating high-temporal-resolution symptom monitoring (e.g., minute-by-minute), particularly in comparison with the current standard of care.

### Digital therapeutics and care (DiGA)

Digital therapeutics (DTx) and app-based care platforms targeting physiotherapy, speech therapy, cognitive training, and medication adherence are rapidly emerging as adjuncts to conventional care for people with PD and related movement disorders. In some healthcare systems—most notably in Germany through the Digital Health Applications (DiGA) program—certain digital therapeutics have achieved regulated reimbursement status, creating one of the first large-scale frameworks for “apps on prescription.” Even though no Parkinson-specific DiGA is listed yet, several comorbidities such as depression, anxiety, fatigure as non-motor symptoms of PD can be targeted by distinct DiGAs (given that the diagnose has been coded). As of early 2026, over 61 DiGAs have been listed, and reimbursement activity has increased substantially, reflecting early adoption at scale within statutory health insurance systems (Schmidt et al. 2024). However, it is important to acknowledge that the current DiGA framework has inherent limitations, particularly the requirement that medical benefits be attributable directly to the app itself. This creates a particular challenge in movement disorders such as PD, where pharmacological treatment, adjunctive therapies, and ongoing physician input remain the principal determinants of clinical benefit.

By enabling patient-centered, structured, home-based therapeutic engagement, digital therapeutics promise to extend specialist interventions beyond the clinic and support high-frequency, low-threshold care delivery. For example, augmented reality (AR) and mobile platforms have been studied as digital gait training tools, demonstrating feasibility and non-inferiority to traditional therapist-led dual-task gait training in randomized clinical trials for postural instability and gait dysfunction—a core PD impairment (Alberts et al. 2023). Similarly, feasibility studies of home-based AR rehabilitation programs show high patient adherence and acceptability, suggesting that engaging digital interfaces may help sustain participation in therapeutic exercises (Geerse 2025). Smartphone-based speech-language therapy applications have also been piloted, indicating potential for remote delivery of voice and articulation exercises, though evidence remains early and heterogeneous (Lee et al. 2025). Despite these promising developments, robust evidence for long-term clinical benefit in PD remains mixed and limited. A recent narrative review points out that many digital therapeutic interventions are underdeveloped and underutilized, with limited rigorous data on their impact on core motor and non-motor outcomes, and often lacking large-scale, long-term randomized trials (Ellis and Earhart 2021). Moreover, although Germany’s DiGA reimbursement model has facilitated access and rapid uptake, systematic evaluations reveal that the clinical evidence base behind many reimbursed DiGAs is still emerging, with concerns about study bias, reliance on patient-reported measures, and a paucity of objective outcome data (Sippli et al. 2025). Integration into routine outpatient workflows also remains modest. Most digital therapeutics operate as stand-alone solutions, poorly interfaced with electronic health records or clinical care pathways, which can limit clinician engagement with therapy usage and outcomes. This gap risks relegating clinicians to passive observers of patient-generated data rather than active interpreters and integrators of digital therapeutic insights. Clear implementation strategies as hybrid care solutions, interoperable data flows, and clinician education are therefore essential to realize the full potential of digital therapeutics in structured outpatient care. Additionally, ongoing policy debates highlight the need for stronger evidence requirements and outcome-based reimbursement models to ensure that digital therapeutics deliver measurable health benefits that justify their integration and cost (Schmidt et al. 2024). In summary, digital therapeutics and DiGA programs represent a transformative step toward personalized, accessible therapeutic care for movement disorders, but their long-term clinical value and integration into everyday practice will depend on rigorous evidence generation, reimbursement models aligned with real-world impact, and seamless clinical embedding.

### Integrated hybrid care networks and electronic patient record

Integrated and digital supported care ecosystems aim to connect diagnostics, therapy, monitoring, and documentation across care sectors, leveraging digital technologies to support more efficient and patient‐centered care—often described as hybrid or managed care. These systems facilitate information flows between general practitioners, specialists, therapists, hospitals, and patients, enabling continuity of care that transcends traditional clinical encounters. Research on digital information ecosystems highlights that seamless information generation, transmission, and exchange along a patient’s care pathway can enhance coordination and reduce gaps in clinical workflows, although challenges remain around interoperability and data silos (Chen et al. 2024). Electronic patient records (EPRs) are fundamental to integrated care networks. When interoperable, EPRs allow health information to be shared and accessed across organizations in a standardized manner, supporting both clinical and administrative tasks. Crucially, the widespread adoption of interoperable EPRs and national electronic health records is essential for digitally enabled care. However, efficiently organizing, extracting pertinent information, and coordinating care for individual patients and their multidisciplinary teams necessitates additional triage, management platforms, and algorithmic support to streamline clinical workflows and facilitate decision-making (Karottki et al. 2025; Sun et al. 2026). Research indicates that interoperability—defined as the seamless exchange and joint use of data across disparate information systems—can significantly enhance medication safety and workflow efficiency, although the degree of benefit depends on factors such as implementation quality, technological maturity, and organizational context.

Patient and caregiver perspectives further emphasize that lack of interoperability negatively impacts care quality. For example, patients often experience fragmented information flows when EPR systems do not communicate effectively across care settings, leading to errors and increased reliance on manual workarounds (Paccoud et al. 2024b). Improvements in interoperability, including better privacy and security safeguards, were identified by patients as necessary to enhance safety and experience (Li et al. 2024). Interoperable EPRs require standardized data formats and communication protocols. Standards such as HL7 FHIR, SMART on FHIR, and SNOMED CT are increasingly adopted to facilitate data exchange between clinical systems, wearables, digital therapeutics, and decision support tools, forming the technical foundation of hybrid care networks (Prabhu et al. 2025). However, adoption remains inconsistent due to variation in technological maturity, cost constraints, and regulatory environments (Ambalavanan et al. 2025). In many countries, national strategies and policy frameworks are advancing integrated EPR systems as part of digital health transformation initiatives. For instance, recent efforts in several OECD countries illustrate a growing exchange of electronic clinical records across care providers, with goals of national‐level interoperability and coordinated care delivery (OECD 2022). Despite this progress, governance challenges persist. Dependence on proprietary platforms can limit data portability and raise concerns over data ownership. Clear data governance structures, standardized interoperability frameworks, and robust privacy protections are essential to maintain trust and maximize the clinical value of hybrid care networks (Hartenstein et al. 2025).

### Digital health literacy and patient acceptance

The success of digital outpatient care depends critically on patient competence, motivation, and trust (Smith et al. 2025). Digital health literacy—the ability to access, understand, evaluate, and use digital health information—has emerged as a crucial determinant of whether patients can fully benefit from digital services. Many patients, particularly those with movement disorders, are elderly, cognitively impaired, or socially vulnerable, which can limit their ability to navigate digital tools effectively. Studies indicate that a significant proportion of adults exhibit low digital health literacy, especially in using mobile health apps and online portals, and this is strongly associated with age, socioeconomic status, and general health literacy. Low digital health literacy not only hampers access to care but also diminishes patient trust and engagement with digital services (Srinonprasert 2024). Acceptance of digital health technologies among older adults is influenced by multiple factors. Facilitators include perceived usefulness and ease of use, prior positive experiences with technology, and encouragement from healthcare professionals, which align with established models of technology acceptance. Conversely, barriers such as low digital skills, technical and usability challenges, privacy concerns, and perceptions of impersonal care can significantly reduce uptake (Aoun et al. 2025). These challenges are compounded in patients with movement disorders, who may face motor difficulties, cognitive impairments, and higher overall care needs, making digital interactions particularly demanding. Socially vulnerable populations encounter additional obstacles, including limited prior exposure to technology and heightened distrust of digital platforms (Kim et al. 2025). Given these barriers, outpatient clinics increasingly assume roles beyond traditional care delivery, acting as educators, mediators, and interpreters of digital health information. As educators, clinics provide tailored instruction and support to enhance digital literacy, such as guided tutorials, one-to-one training, and integration of family caregivers. As mediators, they bridge patients and complex digital ecosystems, adapting technology to patient capabilities and fostering trust in digital care. Finally, as interpreters, clinics help patients make sense of health data generated by digital tools, facilitating informed decision-making and empowering patients in their care. To improve access and acceptance, digital tools should be designed with accessibility in mind, incorporating intuitive interfaces, larger text, multi-modal input/output, and features supporting cognitive and motor limitation (Wilson et al. 2021). Clinics should provide tailored educational programs, leverage support networks, and maintain dual pathways for care to ensure that both digital and traditional services remain available, reducing the risk of exclusion and promoting equitable health outcomes (Theobald et al. 2025).

### Prevention and prediction

Digital technologies are increasingly being applied to the prevention and prediction of disease, targeting prodromal phases through the analysis of subtle motor, sleep, speech, and autonomic signals. These approaches hold significant promise for early intervention and for enriching clinical trials by identifying individuals at risk before overt symptoms appear. By enabling earlier detection, predictive digital tools could allow interventions that slow disease progression or improve symptom management. However, the use of predictive risk information raises profound ethical, psychological, and societal concerns. For many conditions, including neurodegenerative diseases, established disease-modifying therapies are limited or nonexistent. Providing individuals with risk information under these circumstances may inadvertently cause harm, including increased anxiety, stigmatization, or overmedicalization. This underscores the need for careful consideration of the ethical, legal, and social implications (ELSI) of predictive technologies, particularly prior to diagnosis, to safeguard patient autonomy, psychological well-being, and social equity. Proactive strategies are necessary to mitigate potential harms. These include the development of clear guidelines for risk communication, integration of counseling and support services, and engagement with patients, caregivers, and broader society to navigate the implications of predictive digital health tools responsibly. In particular, the development and integration of digital services into everyday healthcare procedures requires a more profound public-patient-involvement (PPI) when codesigning applications and hybrid servicdes—including both, patients and healthcare professionals (Davies et al. 2024; Loisel et al. 2025). By addressing these ethical, psychological, and societal challenges upfront, digital prediction and prevention strategies can maximize benefits while minimizing unintended consequences. Future prevention and prediction frameworks should not rely solely on digital phenotyping but increasingly integrate biological and molecular biomarkers. Oxidative stress markers, inflammatory and immunological signatures, α-synuclein-related fluid biomarkers, neurofilament light chain, genetic risk profiles, and advanced imaging biomarkers may substantially enhance predictive validity when combined with longitudinal digital motor and non-motor data streams. Such multimodal disease modeling may improve early stratification of prodromal syndromes and allow more biologically meaningful personalized intervention strategies.

## Roadblocks to implementation

### Data and interoperability

In the past years, devices like smart phones and smart watches have become natural companions in modern daily life. These devices are equipped with accelerometers and gyroscopes and are therefore theoretically able to track all kinds of movements. Thus, many people are already equipped with devices that could deliver additional information about their movements. Smart watches can track arm swing while walking or presence of tremor. For example, the software StrivePD that uses the sensors of the Apple Watch is cleared by the FDA (Larkin 2022). Neptune, a CE-marked software as a medical device, processes raw motion data from Garmin smartwatches via its AI to provide daily continuous motor fluctuations in free-living environment (Pfister et al. 2020; Sander et al. 2025). Furthermore, there are other dedicated devices with FDA approval (e.g. Personal KinetiGraph, Kinesia 360, STAT-ON, PDMonitor) that can be used to record PD symptoms episodically (Cox et al. 2024; Rodríguez-Martín and Pérez-López 2024). However, many of these devices use their proprietary applications and algorithms to translate raw data from the devices’ sensors to movement patterns (Moreau et al. 2023). In addition to the abovementioned devices, there are a lot of other devices available. A recent systematic review reported that there were 50 different devices available to monitor PD symptoms that were used at 20 locations different body (Packer et al. 2025). Furthermore, different devices use different interfaces for data export, and it is impossible for individual health care professionals or centers to support the broad spectrum of the devices that are available. Therefore, to increase overall acceptance and usability in clinical routine, greater standardization in the reporting of sensor configurations, placement, and algorithmic processing pipelines—as well as transparent validation against clearly defined clinical use cases—would be desirable, while still allowing flexibility for innovation and use-case–specific optimization. Moreover, a data standard would also facilitate to import data into clinical documentation systems and would make data from wearables easier usable not only for general neurologists in daily care, but also for movement disorders specialists in specialized centers or for clinical research. In this regard, open-source algorithms could help to increase usability (Timmermans et al. 2025). However, these also cannot be used in a device-independent way, but are restricted to devices that support the use of open-source algorithms. Even though some studies showed that data from proprietary devices could be integrated into open-source environments, the integration required software and informatics skills that are not available for general users (Abedian et al. 2025). The use of multiple devices (e.g. on different body positions) would need accurate time stamps and synchronization, in order to provide a complete dataset, which is challenging in itself (Izmailova et al. 2024).

### Usability, acceptance and tolerance

Another roadblock is the usability, acceptance and tolerance of digital devices. While some devices, such as the Apple Watch, have a broad user base anyway, other solutions require additional devices. A study investigated the tolerance towards three different worn devices. Of all patients that were offered to wear a device, ~ 70%, agreed. After a one-week experience, depending on the device between 14 and 87% of the patients that used a device, replied that they would wear it for more than one week (Rookes et al. 2025). This shows that the tolerance to wear a dedicated additional device over a longer time is low, dependent on the device. Others also found that the acceptance to wear dedicated devices is lower, when multiple devices are needed (Izmailova et al. 2024). Furthermore, comfort is an important factor for the acceptance of wearable devices (Ma et al. 2025). Regarding this, single-sensor systems are better suited for long-term monitoring (Packer et al. 2025).

However, one should keep in mind that single-handed worn devices such as smart watches can only provide data from one half of the body and therefore cannot fully reflect the patients’ state of movement. This limitation may, however, be partially mitigated by suitable algorithms capable of inferring global movement patterns from unilateral sensor data. Since most people wear a watch on one preferred hand, the symptoms on the other side would only be insufficiently reflected. Therefore, in studies that investigated wearables patients were required to wear the wearable device on the more-affected side (Hadley et al. 2021; Adams et al. 2024; Ma et al. 2025). However, with low acceptance for additional dedicated devices, wearing movement sensors on both sides or changing the side on which a device is worn from the preferred side to the clinically more useful side, could reduce overall acceptance.

### Data safety and trust

Data safety is another important roadblock to implementation of digital tools into clinical routine. Regarding this, the consideration between expected benefit and risk of sharing data (privacy calculus) influences the acceptance of wearable devices (Li et al. 2016; Thapa et al. 2023). Many of the available devices that have the technical capability of tracking movements are produced by companies outside the European Union and therefore trust into data safety might be an issue for some patients, if their personal data are stored and processed on some remote server. This situation could be aggravated by the fact that algorithms how raw data from the devices are translated into a “clinical” observation are not always transparent.

In the European context, successful implementation of digital outpatient infrastructures is strongly shaped by regulatory frameworks such as the General Data Protection Regulation (GDPR), the European Health Data Space (EHDS), and emerging AI governance standards. Compared with the more fragmented, provider-centered HIPAA environment in the United States, European frameworks place greater emphasis on data minimization, explicit consent, transparency, and cross-border interoperability within trusted public infrastructures. This may initially slow deployment but can strengthen long-term patient trust and system-wide adoption. In practical outpatient settings, local on-premise servers, hospital-owned sovereign cloud infrastructures, or certified national health data platforms may enable GDPR-compliant storage and processing while preserving secure interoperability with tertiary referral centers, research consortia, and industry partners.

### Reimbursement and economic incentives

Another roadblock is how usage of data from digital devices could be reimbursed. On the one hand, companies that produce devices that provide data that can be used for health are interested themselves in market share in a market worth billions of dollars (Lodewyk et al. 2025). On the other hand, it is also necessary to reimburse health care professionals that would be the ones interpreting the data provided by wearables to make clinical decisions and talking to the patients about the pros and cons of using digital accessories. In Germany, the Digital Health Care Act issued in 2019 was a first step to rule reimbursement of devices and applications by health insurances (Dittrich et al. 2023). However, reimbursement of hybrid care services is largely missing. However, a nationwide reimbursement pathway for hybrid care models that explicitly includes physician involvement remains largely absent. Instead, physician incentives are currently mostly limited to selective contracts negotiated individually with insurers, a process that is slow, resource-intensive, and difficult to scale. As a result, prolonged contracting timelines may threaten the economic sustainability of many digital health solutions, representing a broader structural challenge of the German healthcare system. The current implementation landscape is characterized by a structural mismatch between scalable reimbursement pathways and disease-specific digital care solutions. As illustrated in Table 1, fully reimbursed digital therapeutics (DiGA) primarily target comorbid conditions, whereas Parkinson-specific hybrid care models—such as ParkinsonGO and Neptune—remain limited to selective contracts with individual health insurers, restricting broader integration into routine outpatient care.

**Table 1:** Practical implementation framework for a digital movement disorders outpatient clinic, including example technologies, clinical workflows, decision triggers, and responsible team roles

Category	Clinical domain	Solution	ICD-10	Reimbursement model	Integration Level
Hybrid care	Gait and mobility	ParkinsonGO	G20	Selective contracts (statutory insurance)	Moderate (real-world pilots)
	Motor fluctuations/mobility	Orbit Neptune	G20	Selective contracts (statutory insurance)	Moderate (real-world pilots)
DiGA (digital therapeutics)	Depression	Deprexis	F32.0–F32.2	Full (DiGA)	Low–moderate
		Edupression	F32.0–F32.1; F33.0–F33.1	Full (DiGA)	Low–moderate
		Novego	F32.0–F32.2	Full (DiGA)	Low–moderate
		Elona Therapy Depression	F32.0–F32.2	Full (DiGA)	Low–moderate
		My7Steps	F32.0–F32.1; F33.0–F33.1	Full (DiGA)	Low–moderate
		Selfapy Depression	F32.0–F32.1; F33.0–F33.1	Full (DiGA)	Low–moderate
	Pain	HelloBetter Chronic Pain	F45.40/.41; M79.7	Full (DiGA)	Low
	Insomnia	HelloBetter Sleep	F51.0; G47.0	Full (DiGA)	Low
		Somnio	F51.0; G47.0	Full (DiGA)	Low
		Somnovia	F51.0; G47.0	Full (DiGA)	Low
	IBS/constipation	Cara Care	K58	Full (DiGA)	Low
	urge incontinence	INKA	N32.8; N39.42	Full (DiGA)	Low
		Kranus Mictera	N39.3–N39.42	Full (DiGA)	Low
	Cognitive impairment	Memodio	F06.7	Full (DiGA)	Low
		NeuroNation	F06.7	Full (DiGA)	Low

### Defining “benefit”

Another open question is, how digital devices could provide an extra value or benefit for patients (Izmailova et al. 2018). Even if available devices would transparently provide information how a detected movement was used to define different movement states (i.e. off-phase, on-phase, dyskinesia, tremor), this information might not necessarily be congruent with the feeling of the patient. This leads to the difficulty of defining a “benefit” from the usage of digital devices (Mejia et al. 2025). For some patients, the usage of a wearable device might even be disadvantageous, if they attribute to much importance to data provided from such a device. Therefore, it would be important to educate patients and health care providers, what a distinct device can and cannot do and not to rely on the “interpretation” of a devise alone. A major practical benefit of digital innovation may lie in the redistribution rather than mere reduction of physician workload. Automated documentation, AI-assisted report generation, and structured pre-interpretation of longitudinal sensor data may reduce administrative burden and free clinical time for patient counseling, caregiver communication, ethical deliberation, and shared decision-making. In this sense, the value of digital technologies should also be measured by their capacity to strengthen the human dimensions of outpatient neurological care while improving efficiency and scalability.

### Interfaces with industry

The acquisition and processing of health data represent a rapidly growing market, and substantial industry investment has been essential for translating digital health innovations into scalable products. Commercial incentives play a legitimate and necessary role in this process, as sustained investment in research, development, regulatory approval, and deployment depends on viable business models. At the same time, data-processing pipelines and interpretive algorithms often constitute valuable intellectual property and are therefore commonly protected as proprietary assets. While this protection is understandable and may be essential to incentivize innovation, it can also limit transparency, interoperability, and opportunities for independent academic validation. Balancing proprietary innovation with sufficient openness for scientific scrutiny and clinical trust therefore remains an important challenge. From the side of companies, there is limited interest in sharing this proprietary information with academic institutions. Nowadays, large companies can conduct their own trials to get approval for their devices as medicinal product (e.g. Apple Heart Study; Perez et al. 2019). With this in mind, academic institutions risk losing leadership in judging medical information. Therefore, open and transparent collaborations between companies that produce devices or provide services that can track movements on the one hand and health care professionals that treat patients on the other hand are needed.

### Clinical guidelines and approval

Guidelines describe a standard of care and give guidance how to treat patients “correctly. While specialists might have the experience to deviate from guidelines in complex or difficult cases, guidelines help less experienced health care professionals to keep a standard. With new therapies emerging, guidelines are regularly updated. Nevertheless, the process of developing or updating guidelines is time-consuming. Therefore, it may take years until new therapeutic or diagnostic options are included in guidelines. In a field that is developing rapidly, it is challenging to keep guidelines up to date. Nevertheless, in some countries, there are recommendations for distinct devices to monitor PD symptoms (https://www.nice.org.uk/guidance/htg657). Furthermore, the process of approval involves many authorities and can also take a long time, and approval is primarily based on safety and not on clinical usability (Lodewyk et al. 2025). In contrast to that, the market for digital devices is evolving extremely fast.

## Roadmap to implementation

### Ethical considerations as a precondition

One of the central questions is not only what is technically possible, but what should be implemented in routine care. Patients have a right not only to know, but also not to know. Predictive information—based on digital health metrics which are required to be standardized by thorough assessment of their content validity, reliability, and responsiveness—must be coupled with counseling, informed consent, and psychosocial support. The treating neurologist in an outpatient clinic or the practice setting remains the key guide within an institutional environment for ethical governance of digital medicine in PD. This role is especially challenged by one of the major aims in PD, i.e. the identification of patients in the very early, prodromal (asymptomatic, non-motor, or early motor) stages which can span decades, by use of personal digital health technologies and community-based point-of-care diagnostic instrumentation (Khanna et al. 2025) If such a recognition of potential early signs and symptoms might on the one hand be of benefit by early interventions—but causally modifying drugs in such very stages do not exist yet—or might on the other hand fatally change a given person´s view on her/his further life including lifetime decisions is a reaction with high interindividual variability. In the field of preclinical disease detection like in PD, e.g. based upon subtle voice modifications, intermittent postural tremor or reduction of walking distances, the ethical aspects of the active decision not to want to know might be underestimated in the use of digital health technologies (Cohen 2025).

### The human factor: the neurologist as communicator

Despite digitalization, the need for human emotional support, trust, and shared decision-making will still be the basis of patient care, perhaps even increase rather than decrease in front of the background of the variety of technical opportunities. Such technical tools are widespread and manifold, as examples, they address motor function in PD and other neurodegenerative parkinsonism (Bacanoiu et al. 2025) and specific clinical features like freezing of gait (Siragy et al. 2025) or speech alterations (Kang et al. 2025) Tools have been developed for the digital assessment of the Unified PD Rating Scale (Piro et al. 2016). For PD assessment and management, a rapidly evolving, immense multitude of wearables is available which is not evaluable by the single neurologist any more (Bhalala and Mansoor 2025; Farabolini et al. 2025; Irani et al. 2025). Neurologists will increasingly assume the role of interpreters of complex digital data and act as communicators of uncertain probabilistic information. Especially when the digital tools are driven by artificial intelligence (AI)-based components, further work is needed to improve the validation processes and enhance clinician involvement for real-world validation and clinicians` input to refine AI integration in clinical settings, ultimately advancing patient-centered outcomes in PD care, with the aim of preserving the integrity of the patient-physician bond (Malaguti et al. 2025).

### Institutional transformation of the outpatient clinic

A recent review by the MDS Technology Study Group summarized that digital health technologies are increasingly achieving high levels of evidence and regulatory recognition in PD and are in the process of being integrated into pharmacological trials for PD, despite challenges in validation, usability, and equity so that patient-centered innovation highlight a clear trajectory toward the development of digital tools for personalized patient care (Virameteekul et al. 2025). Efforts must be taken to ensure that digital health technologies in PD capture clinically relevant, patient-centered outcome measures that are comparable to conventional rating scales and consistently discriminate disease progression, in order to advance patient care in a digital environment. It will be of utmost importance to explore how AI-based tools might be integrated into that process which might be used to translate clinical knowledge into algorithms which are then being applied to enhance early detection of PD symptoms and to monitor patients´ longitudinal clinical status through a combined and perhaps more comprehensive assessment of motor and non-motor manifestations of the disease (Rom et al. 2025). It has been argued in that context that, by capturing dynamic features which can be missed by traditional patient examination, AI-based assessment tools might contribute to a paradigm shift in the long-term management of PD, with broad relevance across neurology, digital diagnostics, and public health innovation (Marano et al. 2025). Future digital clinics will require new professional roles, including digital care coordinators, data nurses, and telemedicine IT specialists. Hybrid care models will combine continuous remote monitoring with targeted in-person encounters. Digital triage systems may optimize resource allocation, but only if embedded in clinically sound governance structures. In that context, there is an evolving regulatory landscape for digital health technologies (including but not limited to informed consent and data sharing agreements), and there are ongoing discussions how pre-competitive public private partnerships might advance the regulatory maturity of digital health technology measures for use in clinical routine or, with additional requirements, also in clinical trials (Hill et al. 2025). Key operational steps for translating these concepts into routine movement disorder outpatient care, including triage, longitudinal monitoring, digital therapeutics, and advanced therapy supervision, are outlined in Table 2. In summary, digital transformation is not inevitable in a normative sense. Healthcare systems must define explicit value frameworks that guide which technologies are adopted, under which conditions, and for which patient groups.

**Table 2:** Overview of implementation pathways for digital health technologies in movement disorder care, including regulatory status, reimbursement models, and integration into clinical workflows

Clinical step in outpatient workflow	Clinical objective	Digital tools already available (examples)	Manufacturers/providers (examples)	Data generated	Clinical action/decision trigger	Responsible team member(s)	Implementation challenges
1. Pre-visit digital intake/triage	Early symptom capture, prioritization of urgent patients, structured referral	Symptom apps, online questionnaires, patient portals, speech pre-screening, smartwatch pre-assessment	Apple/Rune Labs (StrivePD), Portabiles, hospital EHR vendors, REDCap/FHIR portals	Tremor history, falls, OFF episodes, speech changes, medication adherence, patient-reported outcomes	Flag rapid deterioration, falls, hallucinations, suspected atypical syndrome → fast-track specialist visit	Digital care coordinator, triage nurse, neurologist	Older patient usability, incomplete questionnaires, EHR integration
2. Continuous home-based motor monitoring	Longitudinal objective motor phenotyping between visits	Smartwatch sensors, IMU wearables, smartphone gait tasks, ambient home sensors	Apple, Rune Labs, Orbit Health, Global Kinetics (PKG), MC10, PDMonitor®, STAT-ON™, Portabiles	Tremor burden, bradykinesia, gait speed, dyskinesia, arm swing asymmetry, sleep mobility	Increase OFF burden > 20%, worsening gait variability, increased falls → medication review	Neurologist, movement disorder nurse, data specialist	Proprietary algorithms, adherence, side-of-body bias, interoperability
3. Automated symptom diary replacement	Reduce burden of paper diaries and improve ecological validity	Passive wearable logging + app-based event tagging	Apple, PKG, Orbit Health, PDMonitor, smartphone app providers	ON/OFF phases, medication response latency, dyskinesia windows	Detect wearing-off patterns → optimize levodopa interval	Neurologist	Patient trust in automated interpretation
4. AI-supported diagnostic stratification	Early differentiation PD vs APS vs ET	Wearables + speech AI + handwriting + imaging fusion	Hospital AI platforms, academic AI pipelines, cloud-based neurology vendors	Probabilistic diagnosis, confidence intervals, progression risk	Low confidence or red flags → DAT-SPECT/MRI/tertiary referral	Neurologist + AI decision support board	Explainability, bias, medico-legal responsibility
5. Hybrid in-person specialist consultation	Combine digital biomarkers with clinical examination	Dashboard visualization platforms integrated into EHR	Epic/Cerner SMART-on-FHIR modules, custom dashboards	Trend lines of motor burden, adherence, digital biomarkers	Therapeutic escalation, DBS evaluation, infusion candidacy	Movement disorders specialist	Time burden, reimbursement for data review
6. Digital therapeutic prescription	Extend physiotherapy, speech therapy, cognition, mood care into home	AR gait training, speech therapy apps, DiGA-compatible apps, VR rehab	DiGA ecosystem, speech app vendors, AR rehab systems	Therapy adherence, voice volume, gait training completion, mood scales	Poor adherence → care-coordinator intervention; objective improvement → continue	Physiotherapist, speech therapist, neurologist	Limited long-term evidence, poor interoperability
7. Automated medication optimization loop	Personalized treatment timing and dose refinement	Sensor-driven medication response algorithms	Proprietary device platforms, future AI-CDS tools	OFF duration, dyskinesia burden, dose–response curves	OFF > predefined threshold → dose timing adjustment suggestion	Neurologist (final approval), nurse practitioner	Liability, guideline compliance
8. Remote advanced therapy supervision	Monitor DBS and infusion therapies safely	Adaptive DBS platforms, infusion dashboards, wearable-linked alerts	Medtronic, Abbott, Boston Scientific, infusion vendors	Stimulation response, dyskinesia, freezing, night mobility	Alert thresholds trigger urgent tele-visit or device reprogramming	DBS nurse, programmer, neurologist	Regulatory barriers, cybersecurity
9. Non-motor digital surveillance	Capture depression, apathy, sleep, cognition	Sleep sensors, speech analysis, digital cognitive tests	Consumer wearables + medical-grade sleep tools	REM sleep disorder markers, mood scores, voice slowing	Trigger neuropsychology, psychiatry, sleep medicine referral	Neuropsychologist, neurologist	Ethical burden of prodromal risk disclosure
10. Network-based integrated care pathway	Cross-sector coordination between GP, specialist, therapist, hospital	Shared EHR, FHIR interoperability, secure messaging, patient app	National EHR providers, FHIR vendors, EHDS-compatible platforms	Shared medication plan, alerts, therapy milestones	Automatic referral to PT/SLT, admission planning, emergency escalation	Entire MDT	Data silos, governance, reimbursement
11. Outcome tracking & quality management	Evaluate effectiveness of digital clinic	Dashboard KPIs, PROMs, health-economic monitoring	Hospital BI platforms, payer dashboards	Falls, hospitalizations, QoL, OFF time, caregiver burden	KPI-based pathway redesign and reimbursement negotiation	Clinical lead, administrator, payers	Lack of hybrid-care reimbursement

The table highlights differences between fully reimbursed solutions, hybrid care models, and self-pay applications, illustrating current fragmentation and system-level challenges

## Vision of a future digital movement disorder outpatient clinic

In a realistic future care model, the patient journey begins well before the first specialist visit, supported by optional digital screening tools embedded in primary care. A practical implementation framework illustrating representative outpatient workflows, currently available digital tools, example manufacturers, responsible clinical roles, and decision triggers is summarized in Table 2. Following diagnosis, patients enter a hybrid care pathway integrating continuous digital monitoring, app-based interventions, and scheduled in-person consultations. Specialized hospitals will remain essential as tertiary centers, supporting primary care through advanced diagnostics, complex therapies, and system oversight. Hospital-based treatment is traditionally structured around clinical examination, diagnostic reasoning, and therapy selection. Emerging digital technologies are fundamentally transforming these processes, which have historically relied on physician expertise and experiential knowledge. Recent technological advances are expected to substantially reshape both outpatient and inpatient care. Future treatment pathways will increasingly favor outpatient management or brief inpatient stays. Most clinical data will be acquired longitudinally in patients’ home environments using networked sensor systems and telemedicine platforms, forming the basis for therapeutic decision-making and indications for inpatient treatment (Lu et al. 2020). A future Parkinson’s clinic could assume large components of diagnostics, therapy planning, and rehabilitation through sensor technologies, artificial intelligence (AI), robotics, and automated workflows, thereby markedly reducing the need for continuous physician presence. The literature already describes key building blocks of such infrastructures, including wearable-based diagnostics, AI-supported decision models, and robot-assisted physiotherapy and gait training (Pedersen et al. 2020). In this model, clinical processes are organized around a continuous digital process chain in which data from wearables, robotic systems, imaging modalities, and patient interfaces converge within a centralized, AI-driven platform. Clinical decisions are supported by validated decision models trained on multimodal data streams (movement, imaging, laboratory parameters, speech, and behavior) with continuous adaptive learning (Pedersen et al. 2020). Medical roles are partially redistributed to an ecosystem comprising AI systems, specialized nursing and therapy staff, and technical professionals (e.g., engineers, data stewards) responsible for algorithm supervision and hardware maintenance (Landers et al. 2021). Governance structures, including ethics committees and safety boards, assume responsibilities traditionally held by physicians, such as prioritization, treatment limits, and approval of novel algorithms (Pedersen et al. 2020). Human expertise is thus concentrated on supervision, exception management, and system design.

### Diagnostics

Diagnostic pathways for Parkinson’s syndromes will differ substantially from current practice through the integration of biomarkers, advanced imaging, genetic analyses, and AI-supported interpretation. The clinical examination remains central, focusing on cardinal motor features (bradykinesia, rigidity, resting tremor) and structured assessment of non-motor symptoms such as hyposmia, REM sleep behavior disorder, and autonomic dysfunction (Bloem et al. 2021; Tanner and Ostrem 2024). AI-assisted MRI analysis and multimodal algorithms further improve differentiation between PD and atypical parkinsonian syndromes (Tolosa et al. 2021). Motor assessment increasingly relies on layered systems of wearable and environment-integrated sensors that continuously capture motor and non-motor symptoms (Serbée et al. 2025). Anamnestic data are collected remotely using standardized digital questionnaires and stored longitudinally. Wearables equipped with accelerometers, gyroscopes, and pressure sensors enable objective quantification of tremor, rigidity, bradykinesia, freezing of gait (FOG), and gait parameters. Systematic reviews demonstrate high sensitivity and specificity for detecting motor deterioration, supporting both early diagnosis and disease monitoring (Rovini et al. 2018; Lu et al. 2020; Biase et al. 2024). Real-time feedback via visual, acoustic, or vibrotactile cues can be triggered during FOG or imminent fall risk, yielding both diagnostic and therapeutic benefits (Rovini et al. 2018; Bikias et al. 2021; Das et al. 2022). In future clinics, wearables are continuously connected to central platforms that automatically detect deviations from individualized baselines and perform diagnostic classification. Complementary systems include body-worn sensors and camera-based or room-integrated monitoring technologies. RGB-D cameras and motion-capture systems enable automated assessment of standardized motor tasks, including UPDRS items (Tamilselvam et al. 2023). Optical systems reliably capture gait, postural stability, and movement speed even in home environments, facilitating outpatient monitoring and therapy (Lu et al. 2020). Human activity recognition (HAR) algorithms integrate these data to assess daily activities and their qualitative execution (Benyoucef et al. 2025). Multimodal AI models combine wearable data, video analysis, voice recordings, cognitive tests, and imaging (e.g., DAT-SPECT, MRI) to support differential diagnosis across movement disorders (Rovini et al. 2018). Machine learning approaches significantly improve diagnostic accuracy, particularly in early disease stages, and increasingly support prognostic modeling (Biase et al. 2024). In clinics with minimal physician presence, these systems provide probabilistic diagnoses, confidence estimates, differential rankings, and recommendations for further evaluation.

### Automated therapy planning

Advances in biomarkers, wearables, AI, and telemedicine enable earlier detection of disease changes, continuous monitoring, and dynamic therapy adjustment. Remote monitoring allows therapy to be closely aligned with patients’ daily functioning and disease progression, while digital platforms facilitate cross-sector care and access to specialized expertise (Vijiaratnam et al. 2021; Wolff et al. 2023; Stocchi et al. 2024). AI plays a central role in therapy planning by integrating multimodal data—including movement profiles, speech, imaging, and biomarkers—to individualize treatment strategies (Reddy et al. 2024; Priyadharshini et al. 2025; Twala 2025). Wearables and IoT systems provide continuous objective data, analyzed via machine learning to guide therapy adjustments and detect complications early (Fröhlich et al. 2022; Giannakopoulou et al. 2022; Wu et al. 2023). Therapy protocols are adaptive and data-driven, updating in real time as new information becomes available (Pedersen et al. 2020). Pharmacological management follows algorithmic control loops informed by sensor-derived measures of mobility, dyskinesia, and OFF phases, enabling optimization of dosage, timing, and drug combinations (Lu et al. 2020). Automated warning systems identify patterns suggestive of impulse control disorders, orthostatic hypotension, or motor fluctuations and initiate predefined adjustments. Execution and monitoring are conducted by trained staff within validated safety boundaries. In future hybrid outpatient care models, therapeutic drug monitoring (TDM) may complement digital symptom monitoring by integrating pharmacokinetic measurements with continuous wearable-derived response profiles. This may be particularly relevant for therapies with narrow therapeutic windows, fluctuating bioavailability, or complex polypharmacy regimens. Combining TDM with AI-supported symptom-response modeling could enable highly individualized dose timing, escalation strategies, and adverse-event prevention.

### Control of technical therapies (DBS, infusions)

Invasive therapies such as deep brain stimulation (DBS) and infusion therapies are integrated into automated care frameworks. AI-based closed-loop systems dynamically adapt stimulation parameters or infusion rates based on neural signals and motor performance, a concept already under active investigation (Krauss et al. 2021). Adaptive DBS (aDBS) employs machine learning to tailor stimulation in real time, enhancing personalization and therapeutic precision (Oliveira et al. 2023). Sensor-derived movement data guide stimulation adjustments to minimize dyskinesia and reduce OFF time. In future clinics, these systems operate largely autonomously, with safety constraints defined by interdisciplinary oversight.

### Non-motor symptoms and digital interventions

Non-motor symptoms, including depression, sleep disorders, and cognitive impairment, are identified using digital biomarkers. Speech and handwriting analyses detect cognitive slowing, apathy, or depressive features, while sleep sensors identify REM sleep behavior disorder and fragmentation. AI-driven systems allocate patients to digital cognitive training, psychoeducation, or sleep interventions, with intensity automatically adapted. These interventions are delivered via apps, virtual reality, or interactive assistants and complement physical rehabilitation.

### Robot-assisted physical therapy

Robotics and automated training environments form the core of motor rehabilitation. Robot-assisted gait systems (e.g., exoskeletons, robotic treadmills) significantly improve gait, balance, motor symptoms, and fatigue. Virtual reality enables customized scenarios combining motor and cognitive demands, with parameters adjusted in real time based on safety indicators and therapy goals. Clinics operate centralized robotics facilities monitored by a limited number of therapists and technicians. Robot-assisted upper-limb systems quantify and train sensorimotor deficits using adaptive, AI-controlled protocols (Tamilselvam et al. 2023). Virtual tasks target speed, precision, and coordination, enabling high-frequency, standardized, and objectively measurable training.

### Sensory cueing, occupational therapy, and cognitive training

Automated cueing systems address freezing of gait through dynamic visual, auditory, and haptic feedback integrated into robotic environments. Occupational therapy is largely automated using smart environments and assistive robotics that simulate activities of daily living and adapt task difficulty algorithmically (Benyoucef et al. 2025). Fine motor skills are trained using digital pens and haptic interfaces, with immediate feedback incorporated into centralized progress tracking. Cognitive and executive functions are assessed using tablet-based tests and VR simulations. AI-generated cognitive profiles guide adaptive training programs embedded in everyday scenarios. Therapists primarily design and curate training modules, while delivery is automated.

### AI-based decision support and progress monitoring

A central decision-support platform spans the entire care pathway, integrating prognostic models that predict disease progression and treatment response (Chen et al. 2025). Patients are stratified by risk profiles, enabling personalized treatment intensity and sequencing. In physician-reduced clinics, these systems assume major planning functions. The platform also manages infrastructure and resources, dynamically adapting treatment pathways based on real-time data. Algorithms optimize scheduling, monitoring density, and allocation of robotics and telemedicine resources, transforming the clinic into a cyber-physical system governed by ethical and legal constraints.

### Limitations, ethical, and legal considerations

Despite their potential, AI systems require rigorous validation, standardization, and ethical oversight (Fröhlich et al. 2022; Deik 2025). Current consensus emphasizes AI as decision support rather than a full replacement for human expertise (Cerquera-Cleves et al. 2024). Challenges include explainability, bias, data security, and system vulnerability. For invasive therapies and high-risk decisions, legal responsibility is likely to remain with human decision-makers. A realistic future model thus combines extensive automation with reduced but essential human oversight.

### Integrated future scenario

Collectively, these elements describe a highly automated Parkinson’s clinic in which diagnostics are continuous and multimodal, therapy planning is adaptive and AI-driven, and rehabilitation is predominantly delivered through robotics, VR, and smart environments. AI-based progression models coordinate and optimize care pathways, while human experts focus on supervision, governance, and exception handling. Reimbursement shifts toward outcome-based models. Within this framework, a clinic with minimal operative medical presence appears scientifically plausible, provided that overarching human responsibility and control are preserved.

## Discussion

The transition toward digital outpatient care carries significant risks, including acceptance of digital transformation amongst healthcare professionals and patients, overmedicalization, widening digital divides, algorithmic bias, and dependency on proprietary platforms. At the same time, the potential benefits— prevention and prediction, earlier diagnosis, personalized therapy, improved continuity and coordination of care pathways, and patient empowerment—are substantial for Parkinson patients and their team of healthcare professional. The central challenge lies in balancing innovation with ethical restraint, technical feasibility with human dignity, and economic sustainability with equitable access. These aspects are in particular relevant for chronic disease management given the multidisciplinary and trans-sectoral care approach in PD.

To address these challenges and maximize the value of digital transformation, patient-centered devices and co-design approaches—including patient and public involvement (PPI)—must be prioritized. Developing new (real-world) evidence methodologies to rigorously measure clinical utility and healthcare quality are essential, ensuring that digital solutions genuinely improve patient outcomes. Alignment with the upcoming European standards such as the European Health Data Space (EHDS), the AI Act, and the Health Technology Assessment Regulation (HTAR) is critical for interoperability, transparency, and regulatory compliance. Introducing national reimbursement concepts for hybrid care solutions, such as the “disease management program” in Germany, can support equitable access and incentivize quality improvements. Robust education programs for healthcare professionals—both academic and non-academic—should be instituted to enhance digital literacy and foster continuous learning. Updated ELSI (Ethical, Legal, and Social Implications) frameworks must be developed in collaboration with professional societies, medical guidelines, and policy strategies to address emerging challenges and safeguard patient interests.

In the digital future of outpatient movement disorder care, the role of the neurologist will increasingly shift from performing isolated diagnostic or therapeutic tasks toward acting as the central interpreter and communicator within complex digital ecosystems. As continuous monitoring, algorithmic/AI-based clinical decision and care management support, and app-based therapeutics generate large volumes of patient data, physicians will need to contextualize these insights, translate probabilistic or AI-derived findings into meaningful clinical guidance, and ensure that patients understand the implications for their health and lifestyle. Digital supported care enables and requires effective communication and interpersonal competencies that will become as critical as technical expertise, allowing neurologists to support shared decision-making, maintain trust in care relationships, and address the ethical and emotional dimensions of predictive or personalized digital interventions. In this environment, clinicians who can balance high-level digital literacy with empathetic patient engagement will be essential to the success of hybrid care models, ensuring that technological innovation complements rather than replaces the human-centered core of neurology.

## Conclusion and call to action

The future digital outpatient clinic for movement disorders will not be determined by technological progress alone but by deliberate clinical, ethical, and political choices. Clinicians must actively engage in digital innovation to ensure medical relevance. Decision-makers must establish sustainable reimbursement structures and interoperable infrastructures. Policymakers must provide regulatory clarity and ethical safeguards. Without such coordinated action, digitalization risks increasing fragmentation rather than improving care. With it, digital medicine offers the chance to fundamentally improve the long-term management of movement disorders while preserving the human core of neurology.

## Supplementary Information

Below is the link to the electronic supplementary material.

Supplementary Material 1

## Data Availability

No datasets were generated or analysed during the current study.
